# Involvement of circulating soluble HLA-G after liver transplantation in the low immunogenicity of hepatic allograft

**DOI:** 10.1371/journal.pone.0282736

**Published:** 2023-03-10

**Authors:** Bastien Le Floc’h, Nathalie Costet, Nicolas Vu, Pénélope Bernabeu-Gentey, Charlotte Pronier, Pauline Houssel-Debry, Karim Boudjéma, Virginie Renac, Michel Samson, Laurence Amiot

**Affiliations:** 1 Service de Chirurgie Digestive, Inserm, EHESP, IRSET (Institut de Recherche en Santé, Environnement et Travail) - UMR_S 1085, CHU Rennes, Univ Rennes, Rennes, France; 2 Inserm, EHESP, IRSET (Institut de Recherche en Santé, Environnement et Travail) - UMR_S 1085, Univ Rennes, Rennes, France; 3 Pôle de Biologie, Service de Virologie Générale et Rétrovirologie, Inserm, EHESP, IRSET (Institut de Recherche en Santé, Environnement et Travail) - UMR_S 1085, CHU Rennes, Univ Rennes, Rennes, France; 4 Service des Maladies du Foie (SMF), Inserm, EHESP, IRSET (Institut de Recherche en Santé, Environnement et Travail) - UMR_S 1085, CHU Rennes, Univ Rennes, Rennes, France; 5 EFS Rennes, Laboratoire Histocompatibilité-Immunogénétique / Immunologie Leuco-plaquettaire (HLA/HPA), Rennes, France; 6 Pôle de Biologie, Laboratoire de Cytologie-Cytometrie en flux Inserm, EHESP, IRSET (Institut de Recherche en santé, Environnement et Travail) - UMR_S 1085, CHU Rennes, Univ Rennes, Rennes, France; University of Missouri, UNITED STATES

## Abstract

Graft rejection is a critical risk in solid-organ transplantation. To decrease such risk, an understanding of the factors involved in low immunogenicity of liver allografts could potentially make it possible to transfer this tolerogenic property to other transplanted organs. HLA-G, a natural physiological molecule belonging to the Human Leukocyte Antigen class (HLA) Ib family that induces tolerance, is associated with fewer rejections in solid-organ transplantation. In contrast to HLA-G, HLA antigen incompatibilities between donor and recipient can lead to rejection, except in liver transplantation. We compared HLA-G plasma levels and the presence of anti-HLA antibodies before and after LT to understand the low immunogenicity of the liver. We conducted a large prospective study that included 118 patients on HLA-G plasma levels during a 12-month follow-up and compared them to the status of anti-HLA antibodies. HLA-G plasma levels were evaluated by ELISA at seven defined pre- and post-LT time points. HLA-G plasma levels were stable over time pre-LT and were not associated with patient characteristics. The level increased until the third month post-LT, before decreasing to a level comparable to that of the pre-LT period at one year of follow-up. Such evolution was independent of biological markers and immunosuppressive treatment, except with glucocorticoids. An HLA-G plasma level ≤ 50 ng/ml on day 8 after LT was significantly associated with a higher rejection risk. We also observed a higher percentage of rejection in the presence of donor specific anti-HLA antibodies (DSA) and an association between the increase in HLA-G plasma levels at three months and the absence of DSA. The low immunogenicity of liver allografts could be related to early elevated levels of HLA-G, which lead, in turn, to a decrease in anti-HLA antibodies, opening potential new therapeutic strategies using synthetic HLA-G proteins.

## Introduction

The transplantation of solid organs often represents the last therapeutic option for advanced diseases. The most frequent complications are acute or chronic rejection, leading to acute or chronic allograft dysfunction and subsequent graft fibrosis. To prevent such complications, immunosuppressive therapy is required for life but is itself responsible for numerous complications, such as recurrent cancer, new malignancies, and opportunistic infections [1]. Among the various causes of allograft rejection, Human Leukocyte Antigen (HLA) incompatibility between donor and recipient involving both class I (-A, -B, -C) and class II (HLA-DR, -DP, -DQ) antigens is responsible for the alloresponse, involving both innate and acquired immunity [2].

Concerning liver transplantation, HLA incompatibility is not taken into account in the choice of donor because of the low immunogenicity of liver transplantation (LT) relative to other organ transplants [3]. Indeed, the hepatic graft may confer protection to other co-transplanted organs [4], as also shown in combined liver-kidney transplantation [5]. Intrinsic immunoregulatory properties of the liver explain its resilience to antibody-mediated damage relative to heart or kidney allografts.

One way to reduce the risk of graft rejection in organ transplantation would be to increase immunosuppressive therapies, with all their adverse side effects, or increase the tolerogenicity of the graft, hence, the interest in understanding the mechanisms involved in low liver immunogenicity is to potentially boost and transfer them.

Moreover, the tolerance induced by the liver graft could be explained by the expression or secretion of HLA-G, a natural physiological molecule that induces tolerance. HLA-G is a non-classical class Ib molecule first identified to be expressed at the materno-fetal interface [6] and responsible for the tolerance of the fetus to the maternal immune system. Since then, many studies have shown its dual role, both beneficial in transplantation [7] and deleterious in cancer [8]. Its immunomodulating function results from its suppressive properties on specific immune cells (B and T lymphocytes), innate immune cells (segmented neutrophils and natural killer cells), and antigen-presenting cells (monocytes, macrophages, and dendritic cells) [9]. Its immunomodulatory function differs from that of Ia or classical HLA antigens, which can be explained by its distinct features, which are: **(i)** its low polymorphism, contrasting with the highly polymorphic classical HLA class I and class II antigens, **(ii)** the alternative splicing of its primary transcript, deleting specific exons or retaining introns 4 or 2, leading to four membrane-bound and three soluble isoforms, **(iii)** the stop codon in exon 6, leading to a shorter protein, **(iv)** different regulation of its promoter from other class I genes, and (**v**) its restricted expression to immune-privileged tissues under physiological conditions, contrasting with the wide ubiquitous expression of class I a HLA antigens [10].

In situations of transplantation, HLA-G has been shown to be associated with a lower occurrence of acute and chronic rejection in heart, lung, and kidney transplantation [5, 11, 12].

In liver transplantation, the involvement of HLA-G in immune tolerance differs between studies [13–17] and no clear conclusions can be drawn. Outside of transplantation, HLA-G is also associated with certain liver diseases, as it has been detected in the livers of patients infected with hepatitis C virus (HCV) and shown to be associated with fibrotic lesions [18].

We investigated the relative involvement of HLA-G and anti-HLA antibodies (Abs) in the low immunogenicity of the liver by conducting a prospective study to follow the kinetics of plasma HLA-G levels in 118 patients at various times relative to LT (pre-LT, the day of the transplant (D)1, and D8, D15, month (M)1, M3, and ≥ M12 post-LT). We assessed whether plasma HLA-G levels are associated with HLA class I and class II antibody (Ab) levels before LT, on the day of LT, and one year after, as previously reported for certain situations [19].

## Patients and methods

### Cohort

This was a prospective, monocentric, observational study carried out in the hepato-biliary and digestive service of the Pontchaillou University Hospital (Rennes, France).

#### Ethic statement

Ethical approval to report this case series was obtained from the Institutional Review Board of the CHU Pontchaillou of Rennes (NUMBER 16.47).

Non-opposition to the protocol was obtained for all patients included in the study by the referring doctor, who explained the protocol and provided them with a copy of the information letter.

The inclusion criteria were patients over 18 years of age undergoing a first LT. The exclusion criterion was patients who underwent a multi-organ transplant. The final cohort included 118 patients after applying the inclusion and exclusion criteria. Data collection for the cohort is described in S1 Document in S1 File. Patient characteristics are summarized in Table 1. The biological parameters of the patients at various times of LT follow-up are summarized in Table 2.

**Table 1:** Description of the cohort (N = 118 patients).

Variables	Missing	Mean (± std) or N(%)
**N = 118**		
**Age at transplant (yrs)**	0	57.7 ± 9.4
**Sex**	0	
Male		86 (72.9%)
Female		32 (27.1%)
**Etiology**	0	
Alcohol cirrhosis		83 (70.3%)
Biliary diseases		7 (5.9%)
Non-alcoholic fatty liver disease		5 (4.3%)
Viral cirrhosis		13 (11.0%)
Thrombotic disorders		3 (2.5%)
Rare		7 (5.9%)
**HCC**	2	50 (42.4%)
**MELD**	1	18.1 ± 7.8
**CHILD**	8	109
A		20 (18.3%)
B		32 (29.4%)
C		57 (52.3%)
**CMV+ donor**	1	65
**CMV+ recipient**	0	49
**Death**	0	8 (6.8%)
**Average follow-up time (months)**	0	576.3 ± 182.5
**EAD**	0	31 (26.3%)
**Rejection**	40	17 (21.8%)

HCC: hepatocellular carcinoma, MELD (model for endstage liver disease) is a prognostic score to determine the order of priority for the LT calculated from the rates of creatinine, bilirubin, INR, the dialysis at least twice in the past week and +/- sodium level. It varies from 0 to 40, the highest values indicate the most serious condition. The Child-Pugh score is used to assess the prognosis of chronic liver disease. It is calculated from the level of total bilirubin, serum albumin, INR, the presence and the grade of ascites and hepatic encephalopathy. Severity increases from stage A to C.

CMV: cytomegalovirus, EAD: early allograft dysfunction.

**Table 2:** Kinetics of the biological characteristics of the study cohort (N = 118, 7 visits).

Parameter	Pre-LT	D1	D8	D15	M1	M3	> M12
**AST (IU/l)**							
N	117	118	118	118	118	116	110
Mean ± std	84.39 ± 102.4	1027 ± 1589	56.44 ± 34.52	42.56 ± 44.18	29.11 ± 28.29	27.58 ± 24.67	24.45 ± 12.90
Median (Q1—Q3)	59 (40–86)	431.5 (234–1019)	48 (31–69)	29 (18–45)	21 (17–32)	21 (18–29)	20 (17–29)
**ALT (IU/l)**							
N	118	118	118	118	118	116	110
Mean ± std	64.79 ± 123.9	755.70 ± 976.2	170.20 ± 134.1	82.72 ± 68.57	38.92 ± 40.37	34.31 ± 48.11	244 ± 14.36
Median (Q1—Q3)	36 (26–55)	462.5 (271–817)	136 (82–220)	58.5 (39–101)	25.5 (16–44)	20.5 (15–31.5)	19 (13–31)
**APL (IU/l)**							
N	117	118	118	118	118	116	110
Mean ± std	156.3 ± 107	105.4 ± 883	198.5 ± 130.9	201.9 ± 122.6	166.1 ± 106	133.1 ± 183.3	124.3 ± 66.96
Median (Q1—Q3)	129 (94–181)	78 (59–122)	174 (107–253)	167.5 (106–269)	133.5 (94–201)	88 (68.5–118.5)	108.5 (86–139)
**γGT (IU/l)**							
N	118	118	118	118	118	116	110
Mean ± std	126.3 ± 247.7	105.6 ±127	354.9 ± 270.9	285.1 ± 228.3	201.8 ± 193.8	122.6 ± 206.2	71.56 ± 81.64
Median (Q1—Q3)	62 (34–136)	73.5 (38–121)	281.5 (160–466)	241.5 (136–362)	130 (78–239)	46.5 (26.5–108.5)	43 (24–84)
**Bilirubinemia (μmol/l)**							
N	118	118	118	118	118	116	108
Mean ± std	123.7 ± 163.9	78.53 ± 85.31	68.25 ± 759	38.77 ± 54.48	19.58 ± 22.95	13.97 ± 41.11	10.36 ± 6.82
Median (Q1—Q3)	63 (27–131)	42.5 (22–101)	33.5 (18–95)	17 (10–38)	11 (7–22)	7 (4–12)	8.6 (6–12)
**PT (%)**							
N	117	118	117	117	117	115	101
Mean ± std	49.83 ± 22.75	50.35 ± 16.33	85.34 ± 6.48	80.38 ± 137	84.21 ± 14.87	86.71 ± 19.26	94.77 ± 30.19
Median (Q1—Q3)	45 (33–65)	51.5 (37–60)	78 (70–86)	81 (73–90)	86 (76–94)	91 (79–99)	94 (87–100)
**Albuminemia (g/l)**							
N	117	110	76	72	93	97	98
Mean ± std	33.11 ± 6.71	25.41 ± 4.66	28.3 ± 4.57	32.45 ± 6.13	35.9 ± 5.49	39.96 ± 4.93	41.17 ± 4.62
Median (Q1—Q3)	32.8 (28.6–37)	25.2 (22.6–27.9)	27.7 (24.9–32.2)	31.3 (28.1–35.8)	36.2 (33.4–40.1)	40.5 (38.1–43.1)	42 (39–44)
**Creatinine (μmol/l)**							
N	118	118	118	118	118	116	109
Mean ± std	97.38 ± 83.6	114.8 ± 80	87.74 ± 51.22	95.41 ± 57.79	101.7 ± 50.33	85.7 ± 295	95.58 ± 29.26
Median (Q1—Q3)	78 (59–108)	94.5 (65–139)	70 (60–93)	78 (61–108)	90.5 (70–122)	83.5 (66.5–101.5)	88.5 (74.4–112.8)
**Leukocytes (G/l)**							
N	118	118	118	118	118	115	110
Mean ± std	7.11 ± 3.94	13.23 ± 5.94	9.61 ± 6.51	8.98 ± 5.18	80 ± 3.91	4.88 ± 2.23	5.23 ± 1.96
Median (Q1—Q3)	6.1 (4.7–8.2)	12.5 (8.9–16.1)	8.2 (6.3–10.8)	7.9 (6.1–10.4)	7.2 (5.2–10.5)	4.6 (3.4–6.1)	5.1 (3.9–6.4)
**Eosinophils (G/l)**							
N	118	96	110	103	115	113	110
Mean ± std	0.21 ± 0.18	0.32 ± 06	0.37 ± 0.30	0.16 ± 0.16	0.15 ± 0.15	0.15 ± 0.16	0.14 ± 08
Median (Q1—Q3)	0.2 (0.1–0.3)	0 (0–0)	0.3 (0.2–0.5)	0.1 (0.1–0.2)	0.1 (0.1–0.2)	0.1 (0.1–0.2)	0.1 (0.1–0.2)
**Lymphocytes (G/l)**							
N	118	97	110	103	115	110	98
Mean ± std	1.1 ± 0.59	0.43 ± 0.27	0.84 ± 0.5	0.91 ± 0.52	12 ± 0.64	1.5 ± 0.89	1.52 ± 0.90
Median (Q1—Q3)	1 (0.7–1.4)	0.4 (0.3–0.6)	0.7 (0.5–1.2)	0.8 (0.5–1.2)	0.9 (0.6–1.4)	1 (0.6–1.4)	1.4 (1–1.8)
**IS therapy**							
**Simulect** (N)		45					
**FK (ng/ml)**							
N		53	115	117	117	99	85
Mean ± std		2.88 ± 48	3.63 ± 21	5.77 ± 4	61 ± 4.41	5.79 ± 26	5.71 ± 2.23
Median (Q1—Q3)		1 (1–2.1)	3.5 (2.1–5.1)	5.4 (3.9–7.7)	5.9 (4.4–8.3)	5.7 (4.5–7.1)	5.2 (4.4–6.8)
**MPA** (N)		91	117	95	102	94	73
**CTC** (N)		98	113	118	76	67	9
**Ciclo (ng/ml)**							
N		0	0	9	12	12	11
Mean ± std				280.2 ± 182.9	499.3 ± 349.9	487.3 ± 359.1	648.8 ± 308.7
Median (Q1—Q3)				109 (1–372)	463 (155.5–772)	442 (165.5–613)	521 (419–959)
**Evero (ng/ml)**							
N		0	0	1	3	11	13
Mean ± std				7	4.13 ± 1.62	3.33 ± 2.23	65 ± 2.95
Median (Q1—Q3)					4.4 (2.4–5.6)	2.3 (1.8–5.5)	5.4 (4.7–7.6)
**HLA-G (ng/ml)**							
N	116	117	117	118	118	114	105
Mean ± std	31.9 ± 211	38.49 ± 24.39	48.24 ± 322	59.43 ± 50.84	59.99 ± 40.54	64.99 ± 46.71	36.62 ± 37.60
Median (Q1—Q3)	25.9 (16.8–43.2)	32.6 (21.6–47)	38.1 (27.6–64.2)	48 (31.1–69.2)	51.4 (29–73.5)	50.8 (36.8–80)	24.6 (10.7–52.6)

Clinical and biological parameter of the study cohort were collected repeatedly before LT and at D1, D8, D15, M1, M3, and ≥ M12 after LT. This table shows the evolution of the various biological markers and immunosuppressive treatments (tacrolimus-FK, mycophenolate-MPA, solumedrol-CTC, ciclosporin-Ciclo, everolimus-Evero) over time (number of measurements, mean (std), median (Q1, Q3)).

#### Early allograft dysfunction and rejection

Early allograft dysfunction (EAD) was defined as the presence of one or more of the following previously defined postoperative laboratory analysis values reflective of liver injury and function: bilirubin level ≥ 10 mg/dL on D7, international normalized ratio ≥ 1.6 on D7, and alanine or aspartate aminotransferases > 2000 IU/L within the first seven days [20].

Acute rejection was suspected during follow-up consultations by abnormalities of liver parameters, such as cytolysis, hyperbilirubinemia, suboptimal Tacrolimus levels, and/or clinical signs, such as fever, swelling, cyanosis, and tenderness of the allograft. In such cases, immunosuppressive treatment was increased without a liver biopsy. A liver biopsy was performed only when no clinical or biological improvement was observed after one month and in cases of persistent hepatic disruption, despite an increase in immunosuppression, and was planned at 12 months but could be refused by the patient. Rejection was defined according to the Banff classification [21] after a liver biopsy. Two of the three following criteria were required to define acute rejection: (i) a portal inflammatory infiltrate containing lymphocyte and eosinophil blasts, (ii) subendothelial localization of the inflammatory cells in a portal vein branch, and/or (iii) inflammation of and damage to the small bile ducts.

### Methods

#### Specific soluble HLA-G enzyme-linked immunosorbent assay

Plasma HLA-G levels were determined using the sandwich enzyme-linked immunosorbent assay (ELISA) method on the plasma of patients and a control group of 20 participants, as previously described [22] (S2 Document in S1 File). Soluble HLA-G levels were determined before transplantation, and on D1, D8, D15, M1, M3, and M12 after transplantation.

The timing of the plasma HLA-G measurements followed the timing of the usual clinical follow-up consultations of post-LT patients. These consultations occur at defined intervals: once a week for the first two months, then once every 15 days up to 4.5 months, every two months up to a year, every six months up to three years, and then every year. This schedule of visits makes it possible to detect EAD and rejection. As EAD is expected to occur during the first days, the first follow-ups are routinely organized on D1 and D8; acute rejection (or cellular or early rejection) is very frequent during the first 15 days post LT and before six months. Thus, follow-ups are scheduled for D8, D15, M1, and M3. Chronic rejection occurs later (after three months) and can be assessed at M12.

*Anti HLA-antibody determination*. The detection of anti-HLA IgG class I and class II antibodies was performed with Luminex Flow beads using a panel of color-coded beads coated with purified single recombinant HLA antigens. The HLA antigens were those that are the most frequently found in the general population. Patient serum was incubated with single antigen class I and single antigen class II Labscreen beads (One lambda) according to the manufacturer’s protocol. HLA antibodies bind to the beads, which are labeled with R-phycoerythrin coupled with goat anti-human IgG (One lambda). Data were acquired, processed, and analyzed using the Luminex platform and fluorescence intensity expressed as the mean fluorescence intensity (MFI). MFI values > 500 were considered to be positive for anti-HLA antibodies. MFI values of > 2000 were observed in acute rejection.

Patient sera were routinely collected before transplantation, the day of transplantation, and one year after transplantation.

### Statistical analyses

A detailed presentation of the statistical analyses is available in S3 Document in S1 File.

The study sample is described using standard descriptive statistics: means and standard deviations and medians and interquartile ranges for continuous characteristics and frequencies and proportions for nominal characteristics. The distribution of the biological parameters was tested for normality.

The stability of pre-LT levels of HLA-G within a two month-interval was tested in a dedicated 21-patient sub-sample using a paired t test.

Pre-LT HLA-G level determinants were tested using a multiple linear regression model that simultaneously included the patients’ clinical baseline characteristics.

The effect of immunosuppressive (FK) and corticoid treatment on variations in HLA-G levels between the pre-LT period and D8 was tested in a multivariate linear regression model that simultaneously included the patients’ clinical baseline characteristics.

The individual trajectories of HLA-G levels during the follow-up (up to M12) are graphically represented and were modelled using a quadratic mixed effect regression model for repeated measurements. The patients’ clinical characteristics were added to this model as the main and interaction fixed effects to test their impact on the evolution of HLA-G levels during follow-up.

We used multivariate logistic regression models to investigate whether HLA-G measurements during the follow-up period can predict the risk of EAD and rejection. As EAD is defined by graft dysfunction in the first seven post-operative days, we studied the effect of HLA-G levels on D1 and D8 on the risk of EAD. The risk of rejection was studied for patients who had a biopsy 12 months after LT (N = 76). Various time points for HLA-G measurements were tested separately to determine their role in predicting rejection. We produced ROC curves and their respective areas under the curve (AUC) associated with models including *vs* those not including the HLA-G levels. We compared them using Chi-squared tests to determine whether HLA-G levels significantly contribute to the prediction of the EAD or rejection risk.

Statistical analyses were performed using SAS^®^. The level of significance was set to 0.05.

## Results

### Kinetics of HLA-G plasma levels during the LT procedure

#### HLA-G plasma levels according to the clinical status of LTx recipients

There were no significant differences in the mean levels of HLA-G before LT according to the patient characteristics: aetiology, age, sex, MELD score, the presence of HCC, CMV, or status of the recipient or donor (S1A Fig in S1 File). Moreover, there were no significant differences between healthy control patients and transplant patients in the pre-LT period (S1B Fig in S1 File), regardless of the etiology of their initial liver disease. HLA-G levels of patients with other aetiologies did not differ from those of alcoholic-cirrhosis patients.

#### Kinetics of HLA-G plasma levels during the LT follow-up

**Fig 1:**
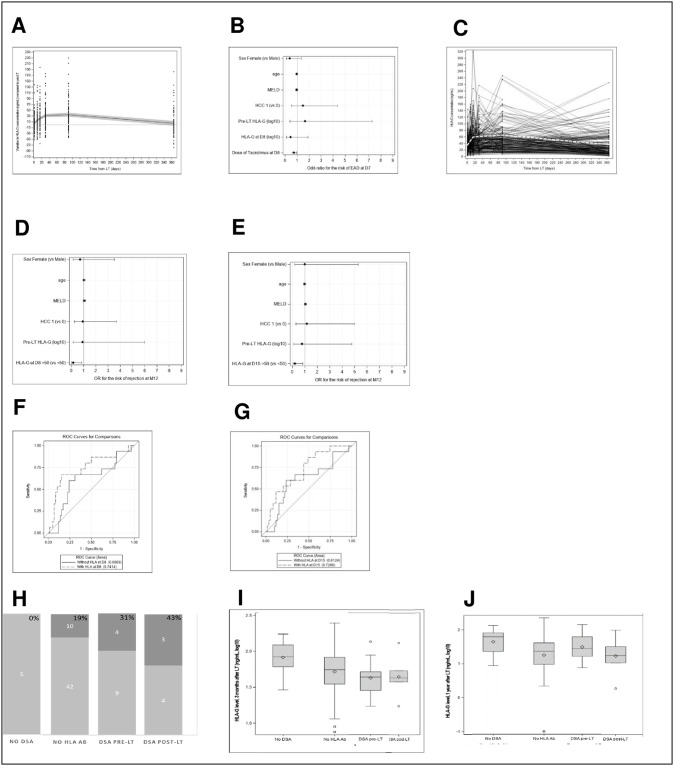
A: Evolution of HLA-G throughout the protocol. Modelling (mixed effect quadratic regression model) of the average kinetics of HLA-G levels over time (N = 118). The Y-axis represents the plasma HLA-G levels (ng/ml) during the follow-up (pre-LT to M12 after LT). Black points represent patients (N = 118). The line represents the mean evolution of HLA-G levels as estimated by the mixed-effect quadratic regression model (confidence interval (95% CI), shown as a grey band). B: Effect of patient characteristics, pre-LT HLA-G levels and those on D8, and tacrolimus treatment on the risk of EAD. Forest plot representing the estimated effect (x-axis) of patient characteristics on the risk of EAD (multivariate logistic regression models). Effects are expressed as odds-ratios (OR) (diamond). A higher plasma level of FK on D8 was significantly associated with a reduced risk of EAD on D7 (OR = 0.74, 95%CI = 0.57; 0.95). C: Individual variability of HLA-G trajectories. Individual kinetics of HLA-G levels over time (N = 118). The vertical axis represents the plasma HLA-G level (in ng/ml). the white dashed line represents the mean trajectory resulting from Loess smoothing. D and E: Forest plots representing the estimated effect (x-axis) of patient characteristics on the risk of rejection (multivariate logistic regression models). Effects are expressed as odds-ratios (OR) (diamond). (D) Effect of patient characteristics and pre-LT HLA-G levels and those on D8 on the risk of rejection (M12) (N = 78 patients). An HLA-G level > 50 ng/mL on D8 was associated with a significantly lower risk of rejection until M12 (OR = 0.15, 95%CI: 0.03; 0.84). (E) Effect of patient characteristics and pre-LT HLA-G levels and those on D15 on the risk of rejection (M12) (N = 78 patients). An HLA-G level > 50 ng/mL on D15 was associated with a significantly lower risk of rejection (OR = 0.20, 95%CI: 0.05; 0.82). F and G: ROC curves for the prediction of transplant rejection (multivariate logistic regression models adjusted for age, sex, MELD, HCC status, and pre-LT HLA-G level). (F) Comparison of models including (full dark line) or not including (dashed line) the HLA-G level at D8 (dichotomised as ≤ 50 ng/mL / > 50 ng/mL) as a predictor. Their respective AUCs are 0.61 (CI: 0.44; 0.78) and 0.74 (CI: 0.58; 0.90). The comparison test of AUCs was nearly significant (p = 0.065), indicating that HLA-G levels at D8 contribute to predicting the risk of transplant rejection. (G) Comparison of models including (full dark line) or not (dashed line) the HLA-G level (≤ 50 ng/mL / > 50 ng/mL) on D15 as a predictor. Their respective AUCs are 0.61 (0.44; 0.78) and 0.73 (0.59; 0.86). The comparison test of AUCs was non-significant (p = 0.19), indicating that HLA-G levels on D15 do not contribute to predicting the risk of transplant rejection. H: HLA antibody (Ab) status and rates of graft rejection. Four Groups can be differentiated: group1: no DSA produced before or after LT, group 2: no HLA Ab present, group 3: DSA present before LT, and group 4: de novo DSA produced after LT (DSA post LT). Rates of graft rejection observed in the four groups of DSA and anti-HLA antibody status among 77 patients with known rejection status. Numbers in the bar chart indicate the frequency of rejection (dark grey bar) and non-rejection (light grey bar). The percentages of rejection are indicated in bold in the upper bar of each antibody status group. The percentage of rejections increases from group 1 to group 4. Group 1 (0%, N = 5), group 2 (19%, N = 52), group 3 (31%, N = 1 3), group 4 (43%, N = 7). I, J: Distribution of HLA-G levels (ng/mL, log10 transformation) in the four groups of patients according to HLA Ab status (G) three months after LT and (H) one year after LT. (I) We found no significant differences in HLA-G levels between groups before three months after LT (ANOVA Fisher tests, all p values > 0.50). At three months, HLA-G levels were higher for patients who did not have DSA (before and after LT) than those who had DSA before and after LT (p = 0.02). No significant difference was found with those who developed DSA after LT (p = 0.07) (1H). (J) At 12 months, these differences were non-significant.

Individual HLA-G trajectories were highly variable in magnitude and shape (Fig 1C). On average, HLA-G plasma levels increased rapidly from D1 to D15, less rapidly until M3, and then decreased between the M3 and M12 visits (p < 0.001). By the 12-month visit, the predicted mean HLA-G plasma level returned to the mean level before LT (Fig 1A).

In multivariate analyses, the global effect of aetiology on the mean levels of HLA-G during the follow-up was significant (p = 0.04), but exclusively attributable to patients with biliary pathologies, who had a significantly higher mean level of HLA-G (55% higher, p < 0.001) than alcoholic-cirrhosis patients (S1C Fig in S1 File). None of the other baseline patient characteristics were associated with HLA-G levels during the follow-up (S1C Fig in S1 File).

#### HLA-G levels and early allograft dysfunction (EAD)/rejection

The rate of EAD on D7 after LT was 26.3% (31/118) in our cohort. An HLA-G plasma level ≤ 50 ng/ml on D15 was observed in most cases of EAD (S2 Fig in S1 File). Baseline patient characteristics, pre-LT HLA-G levels, and HLA-G levels on D8 were not associated with the risk of EAD (Fig 1B).

Only a subsample of 78 patients accepted a liver biopsy after 12 months of follow-up.

In this subsample, 17 (21.8%) showed confirmed rejection (S3 Fig in S1 File). Patients with HLA-G levels > 50 ng/ml on D8 and D15 tended to have a lower risk of rejection (Fig 1C and 1D, S3 Fig in S1 File).

ROC curves derived from the predictive models that included HLA-G levels on D8 or D15 are presented in Fig 1E and 1F, respectively. There was a nearly significant improvement in predicting the risk of rejection with the HLA-G level on D8 (p = 0.06). We found no significant association between the risk of rejection and HLA-G levels at other times of follow-up.

### Evolution of anti-HLA antibodies during the LT procedure

Anti-HLA antibody (HLA Ab) levels were routinely measured at three time points: pre LT, day of LT, 1 year post LT, and were evaluable for 111 patients (Table 3A). When HLA Abs are specific to the donor, they are named donor specific antibodies (DSA). These types of HLA Abs were routinely sought before LT, on the day of LT, and one year after LT. Eighty-five patients were negative for DSA, including 75 patients with no HLA Ab and 10 with HLA Ab other than DSA, and 26 patients were positive for DSA, including 19 patients with DSA pre LT, whereas seven became positive for DSA post LT (DSA *de novo* post LT).

**Table 3:** **A:** Distribution of patients according to the detection of anti-HLA antibodies (Ab), including or not donor specific antibodies (DSA), with their specificity at three time points: pre LT, day of LT, 1 year post LT. **B:** Evolution of DSA pre LT in post LT.

**(A)**	
**Group of patients (N = 118)**	**N**
**No DSA**	**85**
• No HLA Ab	75
• No DSA (pre or post LT)	10
**With DSA**	26
• **DSA pre LT**	**19**
• Anti HLA class I	8
• Anti HLAclass II	8
• Anti HLA class I and II	3
• **DSA de novo post LT**	**7**
• Anti HLA class I	0
• Anti HLA class II	7
**No sera**	**7**
**(B)**	
**Evolution of DSA pre-LT between pre-TH and 1 year post LT (N = 19)**	**N**
**Negativation**	**7**
**Decrease**	**5**
**Stability**	**5**
**Increase**	**2**

Data concerning anti-HLA antibodies (HLA Ab) were collected for 111 patients; 7 serum samples were missing.

Eighty-five patients had no DSA: 75 were negative for HLA Abs (no HLA Abs) and 10 were positive for HLA Abs with no DSA, before and after LT (no DSA). Twenty-six patients had DSA: 19 before LT (Table 3B) and seven became positive for DSA (de novo DSA).

Four types of evolution were observed: the disappearance of DSA for seven patients, a decrease for five patients, stability for five patients, and an increase for two patients.

Indeed, four types of evolution were observed for DSA pre-LT: the disappearance of DSA for seven patients, a decrease for five patients, stability for five patients, and an increase for two patients (Table 3B).

A comparison of the occurrence of graft rejection according to the DSA and HLA Ab status for the available data (77 patients) showed no rejection among patients with no DSA before or after LT (N = 5), 19% rejection among patients without anti-HLA antibodies (N = 52), 31% rejection among patients with DSA present pre-LT and persistent post-LT (N = 13), and 43% among patients for whom de novo DSA appeared post-LT (N = 7) (Fig 1G).

Concerning the distribution of HLA-G in the four groups according to DSA and anti-HLA antibody status, there were no differences in HLA-G levels before three months after LT. At three months, HLA-G levels were higher for patients who did not have DSA (before and after LT) than for patients who had DSA before LT (t-test, p = 0.02). Difference with patients with de novo DSA post- LT was not significant (p = 0.07) (Fig 1I). At 12 months, these differences were non-significant (Fig 1J).

## Discussion

Solid-organ transplantation is an important public health issue due to its increasing frequency, and the monitoring of the delicate balance between the risk of rejection, on the one hand, and the side effects of lifetime immunosuppressive therapy on the other. Thus, understanding the mechanisms involved in the low immunogenicity of the liver graft may make it possible to transfer its tolerogenicity and thus reduce immunosuppressive therapy.

It is well known that liver allografts show immunoregulatory properties and responses to rejection and immune-mediated injuries that are different from those of other organs [3]. The portal and arterial afferent blood input is responsible for endotoxin tolerance and promotes a tolerogenic microenvironment [23]. A number of studies [24, 25] have shown that the low immunogenicity of the liver can also be explained by the systemic release of IL-10 during liver transplantation, in addition to other factors [26, 27]. IL-10 is produced by macrophages of the liver allograft itself and exhibits immunosuppressive properties. In the tolerogenic microenvironment of the liver, natural tolerance-inducing molecules, such HLA-G, may play a role, especially as IL-10 induces a subset of human tolerogenic DCs, called DC-10, that express HLA-G and ILT4 [28]. DC-10 in turn induce regulatory T cells [29].

Here, we describe, for the first time, the kinetics of HLA-G plasma levels before and after LT, allowing us to describe a similar pattern of evolution. We found pre-LT HLA-G levels to be stable and reproducible (S4A Fig in S1 File), as previously reported for heart transplantation [11] and LT [15]. We did not find any significant differences in pre- or post-LT HLA-G levels depending on the aetiology of the liver disease prior to transplantation, as suggested by the study of Moroso et al. [15]. The only covariate that significantly influenced HLA-G levels during follow-up was a biliary etiology. There is little data in the literature on HLA-G expression and biliary cells. Only an association between HLA-G expression in biliary epithelial cells and allograft acceptance in liver-kidney transplantation has been reported [5].

Neither pre- nor post-LT HLA-G levels were associated with severity scores, biological parameters, or the presence of HCC or HCMV. These results differ from those of Baᶊtürk et al. [14]. More surprisingly, despite observing higher HLA-G plasma levels in HCC patients, the difference was not significant, unlike in several other publications [30]. This can be explained by the fact that the criteria for registration on the liver-transplant waiting list are different in France than those of other countries. In France, the alpha-fetoprotein score (AFP score) is used, unlike other countries, which generally use the Milan criteria.

Indeed, patients generally tended to reach similarly high HLA-G levels early after LT, followed by a similar decrease out to 12 months, regardless of the pre-LT HLA-G level (S4B Fig in S1 File). Thus, unlike a previous study, which reported an impact of HLA-G gene polymorphisms on acute rejection after LT [31], we suggest that the initial increase of plasma HLA-G levels is, instead, caused by an extrinsic factor, such as immunosuppressive treatment. Previous studies [32] have shown that tacrolimus (FK) increases HLA-G levels, whereas Levitsky et al. reported a non-significant increase in soluble HLA-G levels, similar to our results [33]. It has also been reported that everolimus is associated with soluble HLA-G expression but not cyclosporin A or mycophenolate. Few patients in our study were treated with everolimus and only from D15. Thus, everolimus was not involved in the increase of soluble HLA-G levels on D8. In our cohort, we did not observe any association between HLA-G levels and the plasma level of tacrolimus during the pre-LT to D8 period, which was characterized by a sharp increase in HLA-G levels for most patients. However, the addition of glucocorticoids was associated with higher HLA-G levels on D8. The upregulation of HLA-G transcription by glucocorticoids is well known and has already been reported in a clinical study [34]. Moreover, corticoids were stopped at M12 for most patients (101/110), which may explain the return to the steady state. HLA-G likely exerts its suppressive properties systemically via its expression in the liver, which is a highly vascularized organ.

Indeed, the mechanisms of HLA-G-mediated tolerance in transplantation have been demonstrated by *in vivo* studies using transgenic mice [35]. It has been shown that the interaction of HLA-G with its receptors induces myeloid-derived suppressor cells, tolerogenic dendritic cells [36], and regulatory T cells and inhibits the function of T8 cytotoxic lymphocytes by down-regulating granzyme B [37], resulting in long-term prolongation of skin allograft survival. However, although the level of evidence confirming the association between less rejection and successful grafting for organs expressing HLA-G is strong, regardless of their type, the role of circulating HLA-G in the blood of the recipient is less clear, as high levels of soluble HLA-G in the blood of liver-kidney and heart recipients [38, 39] are associated with better graft survival, whereas high levels of circulating levels of HLA-G in the blood of lung recipients are associated with acute or chronic rejection [40] Here, we show that plasma HLA-G ≤ 50 ng on D8 or D15 is associated with a significantly higher risk of rejection. This finding is in accordance with those of the study of Naji et al. [39, 41]. Our results differ from those of Moroso [15] who found higher HLA-G in patients with acute rejection during the first two weeks post LT. This discrepancy can be explained by the size of the studied cohort (118 in our cohort versus 35), by the differences of aetiologies and mainly by the difference of detecting antibody to determine HLA-G level. Indeed, Moroso et al. used 56B as detecting antibody in place of anti human beta 2 microglobulin.

Another important result of our study is the demonstration of the role of anti-HLA antibodies. Thus, no rejection was observed among patients with no DSA and a higher percentage of rejection was observed in the presence of DSA than for patients without anti-HLA antibodies. These results are similar to those observed in the transplantation of other solid organs. Indeed, after the transplantation of the liver or other organs, antibody-mediated hyperacute vasculitic rejection can occur in individuals with preformed antibodies against the donor’s major histocompatibility complex (MHC) class I—encoded antigens (DSA). Moreover, the production of anti-donor MHC class I and class II antibodies is also associated with acute and chronic graft damage, usually in the form of transplant vasculopathy [2].

In addition, we show an association between an increase in HLA-G plasma levels at three months post-LT and the absence of DSA. These findings are in accordance with those of the literature, as reported in lung transplantation [40]. In cardiac transplantation, a negative association between HLA-G levels and CD4 staining associated with antibody-mediated rejection has also been shown [42]. The inverse relationship between HLA-G and anti-HLA antibody production is related to the inhibitory role of HLA-G on the function of B lymphocytes, especially antibody secretion.

In conclusion, this study suggests the involvement of HLA-G in the low immunogenicity of the liver, opening new therapeutic perspectives in solid-organ transplantation for the potential use of synthetic HLA-G proteins, which have already been shown to be tolerogenic in vivo [43].

Moreover, this study shows the interest of the follow-up of HLA-G and anti-HLA antibody levels, which are non-invasive markers, to follow graft outcome. Thus, their kinetics, in particular during the early period (D8 and, to a lesser degree, D15 post-LT), may help in identifying suspected cases of EAD (D8) or rejection (mainly D8 and D15), in association with the detection of DSA.

## Supporting information

S1 File (DOCX)
