# Supplementary material for: Involvement of circulating soluble HLA-G after liver transplantation in the low immunogenicity of hepatic allograft
Source: PLoS One. 2023 Mar 10;18(3):e0282736. doi: 10.1371/journal.pone.0282736 (PMC10004504; doi:10.1371/journal.pone.0282736)
Supplement: S1 File — (DOCX) [file pone.0282736.s001.docx]

Table of contents

Supplementary data

Supplementary document 1. Data collection 2

Supplementary document 2. ELISA experiments 2

Supplementary document 3. Statistical analyses 3

References 7

Supplementary Tables and Figures

Supplementary Table 7

Supplementary Figure 1A 8

Supplementary Figure B 8

Supplementary Figure C 8

Supplementary Figure 2 9

Supplementary Figure 3 10

Supplementary Figure 4A 11

Supplementary Figure 4B 11

Legends of supplementary Table and Figures 12

**Supplementary document 1. Data collection**

Clinical, biological and histological information were obtained from the hospital database and database of “Agence de Biomédecine”. Surgical techniques for both procurement and recipient operation were performed according to the usual techniques. After the transplant, the patients were treated according to the reference protocols in the department (Simulect if preoperative renal failure, Tacrolimus (FK) is the immunosuppressive treatment of reference, associated with Mycophenolate (MPA) and corticoids). The transplant indication, primary aetiology, other aetiology, and the presence of Hepatocellular Carcinoma (HCC) were collected from the French Biomedical Agency. The value of Metavir, MELD and CHILD scores used were extracted from the hospital database at the time the patient was registered on the waiting list. At D7 of follow-up, we identified patients with Early Graft Disfunction (EAD) according to the criteria previously described by Olthoff (1) (International Normalized Ratio ≥ 1.6 in day 7, bilirubin ≥ 10mg/dL on day 7, and alanine or aspartate aminotransferases > 2000 UI/L within the first 7 days after transplantation). Moreover, plasmas of 20 healthy subjects obtained from “Etablissement français du sang” (EFS) Rennes were obtained to be used as controls in some of our analyses, after obtaining their agreement according to the rules required by ethic committee.

**Supplementary document 2. ELISA experiment**

**HLA-G**

Plasma patients were obtained from whole blood, taken from EDTA tube and immediately frozen at -80°C, after centrifugation at 1000g, at 4°C. HLA-G specific sandwich Enzyme linked-immunosorbent assay was performed using MEM-G/9 (Exbio, Prague, Czech Republic), and capture by rabbit anti human beta-2 microglobulin (DAKO, Trappes, France) combined with peroxidase enzyme for revelation (minor modification in comparison to previously described (2). After the second antibody, DAKO envision system HRP was added and tetramethylbenzidine/peroxide was used as the substrate^(2)^. To check the stability of HLA-G level before LT, 21 patients of the cohort were tested twice at a 2 month-interval.

**Supplementary document 3. Statistical analyses**

The study sample was first described using usual descriptive statistics: mean and standard deviation, median and interquartile range for continuous characteristics, frequencies and proportions for nominal characteristics. The distribution of the biological parameters was tested for normality and they were log_10_ transformed when necessary (all HLA-G measurements were log_10_ transformed before modelling). The evolution of liver-related biological markers along follow-up was graphically described.

The stability of pre-LT levels of HLA-G within a two month-interval was tested in the 21 patients dedicated sub-sample using paired t-test after log_10_ transformation. Pre-LT HLA-G level variations were studied in a multiple linear regression model including simultaneously patients’ clinical baseline characteristics: sex, age, aetiology, severity of the liver disease (MELD score), the presence of HCC and the HCMV status of the donor and the recipient.

To study the effects of immunosuppressive treatments on HLA-G levels, we first focused on their variations between pre-LT and D8, as they are particularly strong in that period. We implemented multivariate regression models where the HLA-G level at D8 was the dependent variable regressed on the pre-LT HLA-G level, baseline covariates (aetiology, age, sex, CHC, MELD score) and EAD (indicator of a dysfunctioning liver), FK immunosuppressive treatment (dose, continuous variable) and the presence of a corticoid treatment (binary).

Individual trajectories of HLA-G levels along the complete follow-up were then graphically represented and modelled by multivariate non-linear mixed effect regression models for repeated measurements. Patient was considered as a random effect, time (visit) was considered as a fixed effect and coded as a categorical variable (visit number) to fit a potentially non-monotone evolution of the HLA-G during the follow-up. All other characteristics of the patient and his treatment were considered as fixed effects to predict the HLA-G levels along the follow-up. As HLA-G measurements were log_10_ transformed to meet the regression modelling hypotheses, the additive effects of the characteristics on the log_10_-HLA-G levels (estimated as β_j_) have to be interpreted as -fold multiplicative effects on HLA-G measurements (original scale).


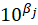

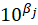


We tested the effect of the pre-LT level on the post-LT levels using a clustering of the patients in 3 categories according to their pre-LT HLA-G levels (< 15 ng/mL, [15-50] ng/mL, > 50 ng/mL). This variable was introduced as a fixed effect in the mixed effect regression models for repeated measurements from D1 to M12. The interaction between pre-LT levels and time of follow-up was included in the model to test differential levels of HLA-G in the post-LT period according to category of pre-LT level.

The results of the mixed effect regression models were represented by figures called Forest Plots. These plots represent the effect of the different patients’ characteristics included in the multivariate models. Stars represent the effects of the variables (noted β_j_), with their 95% confidence interval as lines around. The black vertical bold line represents a null-effect. For quantitative characteristics (such as age, for example), the effect corresponds to the difference in log_10_(HLA-G) effect expected for a one-unit increase in that characteristic (for age, a one-year increase). For qualitative characteristics (such as aetiology, for example), one modality is considered as the reference and is not represented on the plot (“Alcoholic cirrhosis” for the aetiology). For binary characteristics (such as HCC), the reference is the absence of the characteristic. For each modality plotted on the graph, the star (β_j_) indicates the difference in mean log_10_(HLA-G) level expected in patients with this modality, compared to patients with the reference modality. These effects correspond to a -fold variation in the original scale of HLA-G measurements.


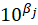

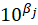


We investigated whether there was a relationship between the level of HLA-G and the risk of EAD. Since EAD is defined by graft dysfunction in the first 7 post-operative days, we studied the effect of levels of HLA-G at D1 and D8. The risk of EAD was modelled using a multivariate logistic regression including baseline characteristics of the patients (aetiology, age, sex, MELD score, CHC), pre-LT HLA-G level and immunosuppressive treatment doses (Tacrolimus and corticoid treatment) at D1 or D8 as predictors.

The risk of rejection was modelled for patients who had a biopsy 12 months after LT (N=76). We used logistic multivariate regression models including the same baseline characteristics of the patients as for EAD risk, the pre-LT HLA-G level and the different post-LT HLA-G levels successively, one after another as covariates. This allowed us testing whether some particular time(s) of HLA-G measurement may be predictive of a later rejection. After visual inspection of HLA-G levels in patients with rejection, we dichotomized the HLA-G level at D8 and D15 using the ad-hoc 50 ng/mL threshold before including it in the regression model. We produced the ROC curves associated with models including the D8 HLA-G or D15 HLA-G levels and computed their respective Area Under Curve (AUC). We compared these AUCs with the one obtained from a model without HLA-G level at D8 or D15, using a Chi-square test. A significant test indicates that the HLA-G D8 and/or D15 levels contribute significantly to the prediction of the rejection risk.

To synthesize the analyses, the individual HLA-G trajectories (pre-LT to M12 visits) were clustered using a k-means algorithm (kml R package). The algorithm aggregates individual trajectories into homogeneous groups. As the number of groups has to be defined a priori, the algorithm was run for 3, 4 and 5 clusters, and rolled 6 times for each number of clusters. The Calinski-Harabatz criterion was used to decide the best partitioning. Then clusters of HLA-G trajectories were described against the clinical characteristics of the patients, using Chi-square tests (categorical variables) or Anova (Fisher test, continuous variables). Analyses were implemented with SAS® and R softwares. Significance level was set at 0.05.

**Supplementary document 4: anti HLA-antibodies determination**

**References**

1 Olthoff, K. M., Kulik, L., Samstein, B., Kaminski, M., Abecassis, M., Emond, J., Shaked, A., and Christie, J. D. 2010. Validation of a current definition of early allograft dysfunction in liver transplant recipients and analysis of risk factors. *Liver Transpl* 16:943.

2 Rebmann, V., Busemann, A., Lindemann, M., and Grosse-Wilde, H. 2003. Detection of HLA-G5 secreting cells. *Hum Immunol* 64:1017.

**Supplementary Table**

|  | **Patients without biopsy** | | | **Patients with biopsy** | | |  |
| --- | --- | --- | --- | --- | --- | --- | --- |
| **Characteristics** | **N** | **mean± std  or %** | **median (Q1-Q3)** | **N** | **mean± std  or %** | **median (Q1-Q3)** | ***p-*value** |
| Age | 40 | 59.3±9.5 | 60.5 (56.0 - 65.5) | 78 | 56.9±9.2 | 60.0 (53.0 - 63.0) | 0.07 |
| MELD score | 39 | 17.6±7.8 | 17.0 (11.0 - 23.0) | 78 | 18.4±7.9 | 18.0 (12.0 - 23.0) | 0.60 |
| Sex |  |  |  |  |  |  | 0.35 |
| Male | 13 | 32.5 |  | 19 | 24.4 |  |  |
| Female | 27 | 67.5 |  | 59 | 75.6 |  |  |
| Aetiology |  |  |  |  |  |  | 0.67 |
| Alcoholic Cirrhosis | 27 | 67.5 |  | 56 | 71.8 |  |  |
| Biliary Disease | 2 | 5 |  | 3 | 3.9 |  |  |
| Metabolic Cirrhosis | 5 | 12.5 |  | 8 | 10.2 |  |  |
| Rare Disease | 1 | 2.5 |  | 6 | 7.7 |  |  |
| Thrombotic Disease | 2 | 5 |  | 1 | 1.3 |  |  |
| Viral Cirrhosis | 3 | 7.5 |  | 4 | 5.1 |  |  |
| HCC |  |  |  |  |  |  | 0.76 |
| Yes | 22 | 55 |  | 44 | 57.9 |  |  |
| No | 18 | 45 |  | 32 | 42.1 |  |  |
| EAD at D7 |  |  |  |  |  |  | 0.27 |
| Yes | 27 | 67.5 |  | 60 | 76.9 |  |  |
| No | 13 | 32.5 |  | 18 | 23.1 |  |  |
| Before LT |  |  |  |  |  |  |  |
| HLA pre-LT | 40 | 33.5±25.8 | 26.9 (16.3 - 43.2) | 76 | 31.1±18.1 | 25.4 (17.0 - 43.2) | 0.98 |
| ASAT pre-LT | 39 | 77.8±74.6 | 55.0 (36.0 - 85.0) | 78 | 87.7±114.1 | 60.0 (45.0 - 94.0) | 0.46 |
| ALAT pre-LT | 40 | 55.2±59.9 | 34.5 (25.0 - 59.0) | 78 | 69.7±146.4 | 37.0 (28.0 - 55.0) | 0.55 |
| PAL pre-LT | 39 | 155.8±127.5 | 118.0 (81.0 - 174.0) | 78 | 156.5±96.1 | 133.5 (98.0 - 183.0) | 0.24 |
| gGT pre-LT | 40 | 138.5±240.6 | 57.0 (26.5 - 131.0) | 78 | 120.1±252.6 | 66.5 (38.0 - 147.0) | 0.40 |
| Bilirubinemia pre-LT | 40 | 114.7±140.3 | 56.5 (24.5 - 125.0) | 78 | 128.3±175.4 | 64.5 (27.0 - 131.0) | 0.83 |
| TP pre-LT | 40 | 52.8±23.6 | 52.0 (34.0 - 69.0) | 77 | 49.2±21.7 | 43.0 (33.0 - 60.0) | 0.43 |
| At 3 months |  |  |  |  |  |  |  |
| HLA M3 | 38 | 64.7±49.1 | 49.7 (35.1 - 82.2) | 76 | 65.1±45.8 | 51.6 (37.2 - 78.3) | 0.88 |
| ASAT M3 | 39 | 28.1±18.4 | 22.0 (15.0 - 37.0) | 77 | 27.3±27.4 | 21.0 (18.0 - 26.0) | 0.55 |
| ALAT M3 | 39 | 32.2±28.7 | 22.0 (13.0 - 42.0) | 77 | 35.4±55.6 | 19.0 (15.0 - 30.0) | 0.67 |
| PAL M3 | 39 | 136.9±164.8 | 88.0 (66.0 - 116.0) | 77 | 131.1±193.0 | 87.0 (69.0 - 128.0) | 0.77 |
| gGT M3 | 39 | 126.6±185.6 | 58.0 (27.0 - 135.0) | 77 | 120.5±217.1 | 46.0 (26.0 - 107.0) | 0.57 |
| Bilirubinemia M3 | 39 | 20.6±65.3 | 7.0 (5.0 - 12.0) | 77 | 10.6±19.7 | 7.0 (4.0 - 12.0) | 0.78 |
| TP M3 | 39 | 80.9±23.8 | 90.0 (71.0 - 97.0) | 76 | 89.7±15.8 | 92.5 (84.0 - 99.0) | 0.08 |
| At 12 months |  |  |  |  |  |  |  |
| HLA M12 | 32 | 35.4±28.5 | 26.4 (16.7 - 48.9) | 73 | 36.4±41.2 | 24.6 (10.3 - 54.9) | 0.44 |
| ASAT M12 | 35 | 23.4±10.5 | 20.0 (16.0 - 28.0) | 75 | 24.9±13.9 | 20.0 (17.0 - 32.0) | 0.69 |
| ALAT M12 | 35 | 20.4±12.6 | 16.0 (12.0 - 28.0) | 75 | 25.7±14.9 | 22.0 (15.0 - 35.0) | 0.04 |
| PAL M12 | 35 | 131.3±72.8 | 101.0 (86.0 - 138.0) | 75 | 121.1±64.3 | 114.0 (85.0 - 140.0) | 0.90 |
| gGT M12 | 35 | 63.1±67.9 | 34.0 (25.0 - 73.0) | 75 | 75.5±87.5 | 46.0 (24.0 - 84.0) | 0.51 |
| Bilirubinemia M12 | 34 | 9.0±4.6 | 8.8 (6.0 - 11.0) | 74 | 11.0±7.5 | 8.4 (6.0 - 12.3) | 0.41 |
| TP M12 | 29 | 102.6±53.5 | 94.0 (92.0 - 100.0) | 72 | 91.6±10.9 | 94.0 (86.5 - 100.0) | 0.85 |

A

C

B

Supp.Figure 1

***
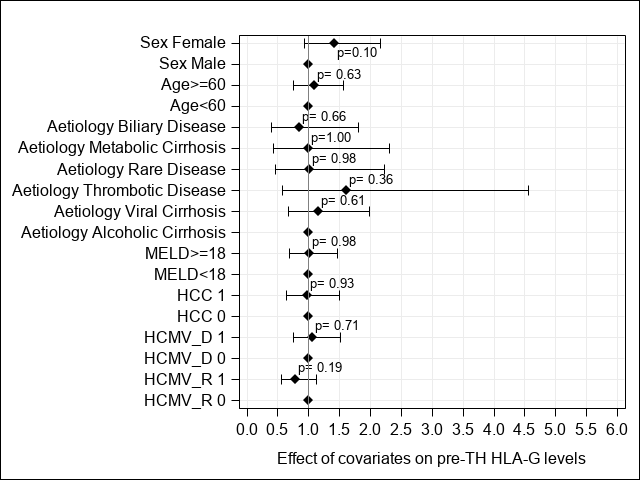
***

***
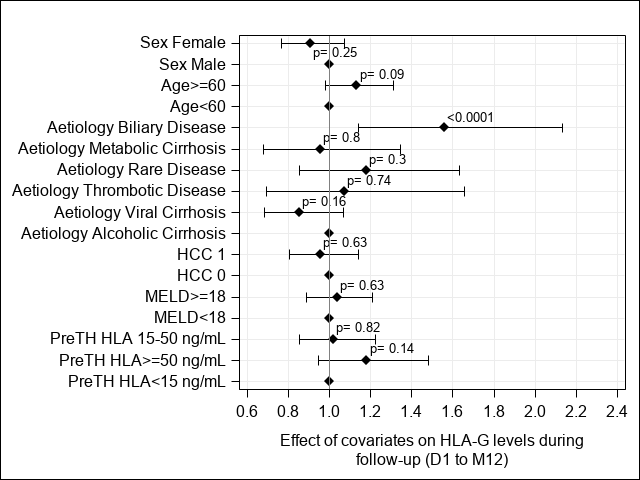
***

Supp Fig.2

Supp. Fig. 3

**Supp. Fig.4A**

**Supp. Fig.4B**


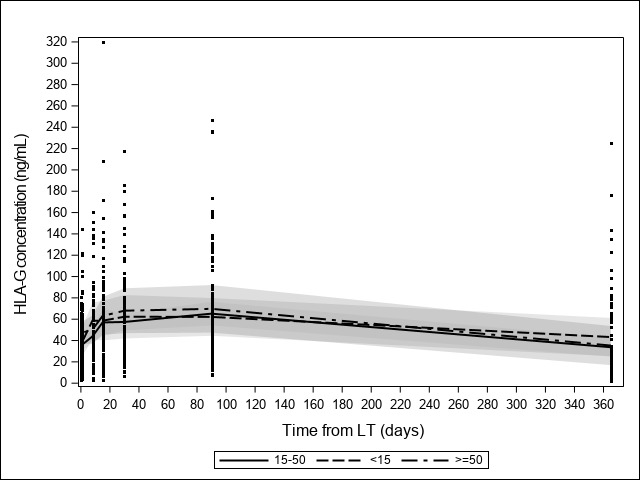


**Legends of Supplementary Table and Figures**

**Supplementary Table**: **Comparison of patients with and without a biopsy at M12.**

Baseline patient characteristics, liver-related biological parameters and HLA-G levels before LT and 3M and 12M after LT. Number of patients, mean (±std) and median (interquartile range) are presented for continuous characteristics, Number of patients and % are presented for categorical characteristics. P-values from Kruskall-Wallis tests for the continuous characteristics, and from Chi-square tests for the categorical characteristics.

**Figure 1 Evolution of HLA-G throughout the protocol**

**(A)** Forest plot representing the effect of the various patient characteristics on the pre-LT HLA-G levels (multivariate linear regression models). For each characteristic, a reference category is chosen and HLA-G levels in the other categories are compared to those in that reference category. Reference categories are “alcoholic cirrhosis” for aetiology, “male” for sex, and “absent” for the other binary characteristics (HCC, HCMV-recipient, and HCMV-donor). An effect equal to 1indicates no effect (vertical grey line), an effect < 1 indicates lower HLA-g levels and an effect > 1 indicated higher HLA-G levels. **(B)** Plot of plasma HLA-G levels versus aetiology. The x-axis corresponds to the various aetiologies and the y-axis to the pre-LT mean plasma HLA-G level (ng/ml). The mean and standard deviation of the plasma HLA-G level is shown for each aetiology. The pre-LT mean of plasma HLA-G levels did not differ according to aetiology nor from those of the control group. **(C)** Forest plot representing the effect of time (visit) and the various patient characteristics on HLA-G levels throughout the follow-up (multivariate non-linear regression model). Reference categories are “alcoholic cirrhosis” for aetiology, “male” for sex, and “absence” for HCC. For the “Visit” (time) variable, the pre-LT visit is the reference. The plot shows that patients with biliary pathologies had significantly higher HLA-G levels throughout the follow-up, compared to alcoholic cirrhosis patients.

**Supplementary Figure2 and Figure 3: Individual trajectories of HLA-G levels in patients presenting an EAD (Fig.2) or a histologically confirmed rejection (Fig.3)**

Individual trajectories of all patients presenting an EAD (sup Fig.2, N=31) or a histologically confirmed rejection (sup Fig.3, N=17) are represented. The x-axis represents the different times of the protocol i.e pre LT (1), D1 (2), D8 (3), D15 (4), M1 (5), M3 (6), ≥M12 (7).The vertical axis represents the level of HLA-G plasma level (ng/ml). In Supp Fig. 2 and 3, the horizontal black line separates respectively patients with an HLA-G plasma level at D15 (4) ≤ 50 ng/ml (upper part) vs patients with an HLA-G plasma level at D15 (4) > 50 ng/ml (lower part). Respectively 17/31 patients with available EAD information and 12/17 patients with available rejection information had HLA-G plasma level ≤ 50 ng/ml at D15 (4).

**Supp Figure 4A: stability of HLA-G levels in pre TH period**

Comparison of HLA-G levels measured twice within 12 months in the pre-LT period (21 patients). There was no significant difference between the means (paired t-test p = 0.98)

**Supp Figure 4B Evolution of the average level (and 95% CI) of HLA-G (Y-axis, ng/mL), stratified by the pre-LT level of HLA-G (3 categories).** The solid curve corresponds to patients with pre-LT HLA-G plasma levels < 15 ng/ml (N = 23), the dashed curve to those with levels of 15-55 ng/ml (N = 75), and the dash-and-dot curve to those with levels > 55 ng/ml (N = 17).
